# Spatial patterns of urbanising landscapes in the North Indian Punjab show features predicted by fractal theory

**DOI:** 10.1038/s41598-022-05906-4

**Published:** 2022-02-02

**Authors:** Thanh Thi Nguyen, Ellen Hoffmann, Andreas Buerkert

**Affiliations:** Organic Plant Production and Agroecosystems Research in the Tropics and Subtropics (OPATS), University of Kassel, Steinstr. 19, 37123 Witzenhausen, Germany

**Keywords:** Environmental sciences, Planetary science, Mathematics and computing

## Abstract

Understanding and governing human settlement patterns is a major challenge of the urban age. While rural settlements emerge as parts of agricultural landscapes, cities typically evolve in economically strategic locations, and over time form hierarchical systems of cities. Purposeful planning and the collective, self-organized behavior of the inhabitants interact in the development of regional settlement patterns. Since self-organizing systems often produce fractal patterns in nature, this study combines approaches of land use science, city ranking, and urban planning under a fractal theory framework, to analyze the settlement system of the Indian Punjab. Scaling levels were defined by discontinuities in the size distribution of built-up areas (Global Urban Footprint), which correlated to population-based classifications (r = 0.9591). Self-similarity across scales was supported by geo-statistical similarity (*p* < 0.05) of distances and angles between settlements of successive classes, and the overall fractal dimension of D_B_ = 1.95. When compared to a modeled Sierpinski Carpet, more than 50% of the settlements met the fractal geometry rules at larger scales. The spatial distribution of small villages, however, deviated, indicating a scale-related shift in organizing principles. Explicitly acknowledging cross-scale relations and self-organisation in regional planning policies may lead to more sustainable settlement structures that are in harmony with natural system properties.

## Introduction

**Figure 1:**
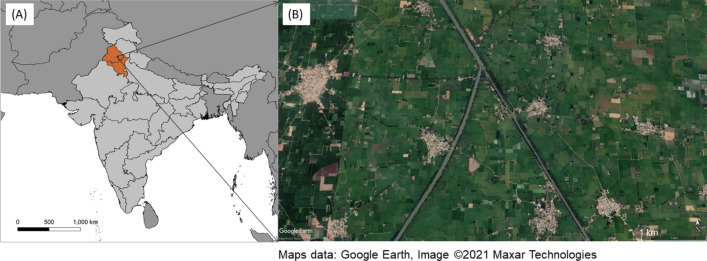
Study region. (**A**) Location map, generated from QGIS 3.16 (QGIS Geographic Information System. Open Source Geospatial Foundation Project. http://qgis.osgeo.org). Map of India (light grey) indicating the North Indian Punjab region (orange) and the location of the exemplary site shown in (**B**). (**B**) Exemplary landscape by satellite view (Google Earth Pro 7.1, accessed 20.09.2021) In this section, small villages are visible amidst agricultural fields, two major waterways supplying the irrigation system, and small streets connecting the villages.

Human activities are increasingly shaping the Earth’s surface. In view of accelerating urbanisation in many regions of the world, there is growing interest in the analysis of settlement patterns fueled by a desire to trace and project urbanisation development over time, derive cause-effect mechanisms, and direct rural–urban transformation towards desired trajectories. Settlement patterns, at a regional or national scale, are usually described by population numbers and densities within administrative units and their geographic distribution, so as to designate rural and urban areas as a basis for development monitoring, policy and planning^1,2^. Many approaches use remotely sensed land cover to analyze urbanisation dynamics at various spatial scales^3,4^. Such studies, however, often focus on particular urban centers with their sprawl over time and do not extend much beyond the respective metropolitan areas^5^. In the this study, we analysed settlement patterns across the entire North Indian Punjab region, a highly anthropogenic landscape marked by decades of intensive irrigation agriculture and a high degree of urbanisation^6^. Agricultural land and housing, along with the connecting infrastructures (streets, railways, waterways), are virtually the only landscape features (Fig. 1). While this is, on one hand, obviously a result of a site-specific history, we aim to determine whether the Punjab landscape may also exemplify some general principles of (self-)organisation which, if properly understood could also apply to other areas of the world operating under similar agro-ecological conditions. Thus, we subjected this human-made settlement pattern to a general, conceptional pattern analysis.

Generally, a pattern is defined by the size and size distribution of its elements, the distances between them, and their spatial arrangements or geometries, such as random scattering, clustering, symmetries, or hierarchies, with some repetitive features across space or scales. Pattern formation may be driven by purposeful planning, by self-organising processes, or by any mix of both.

**Figure 2:**
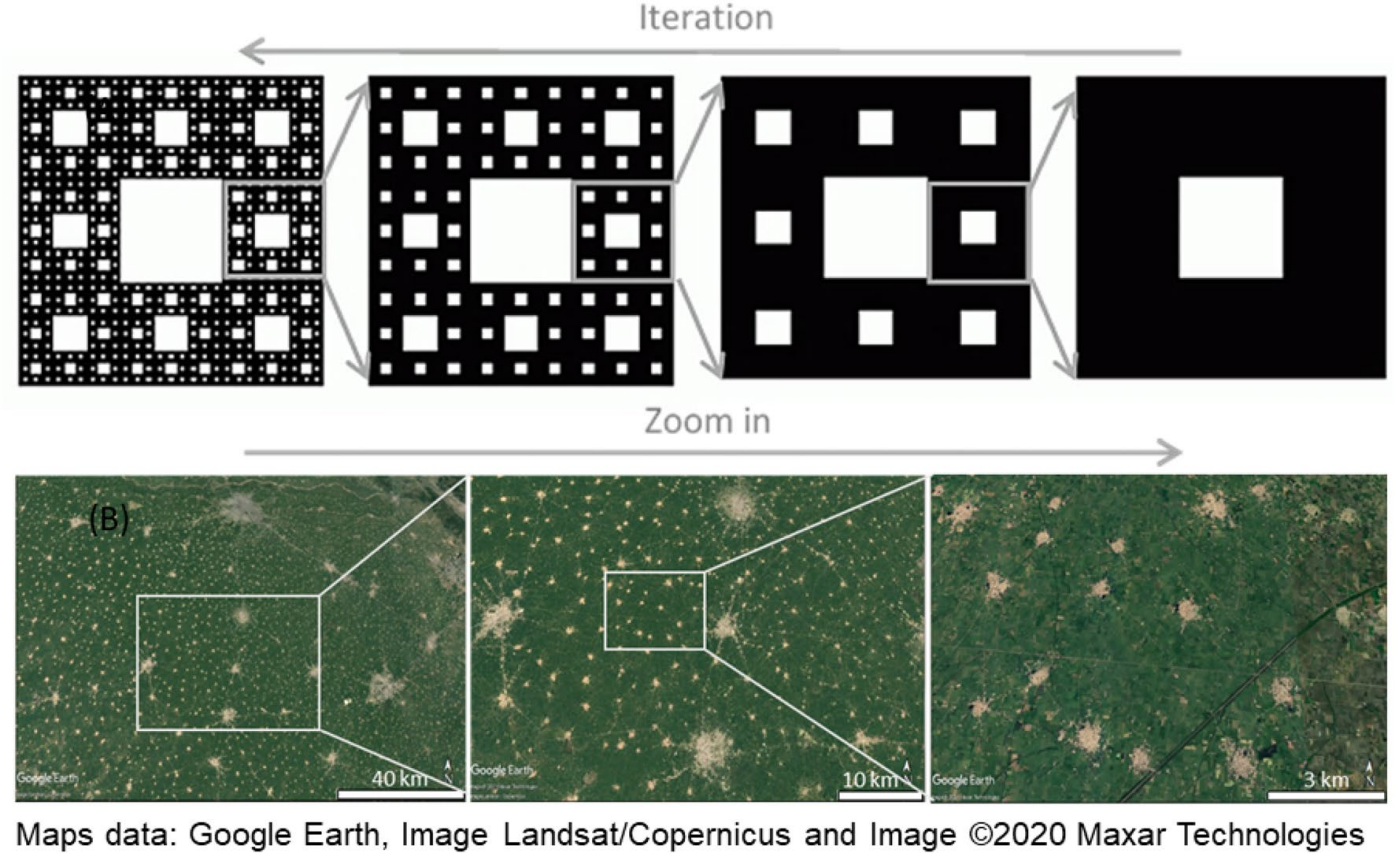
Self-similarity at different scales. (**A**) Sierpinski Carpet fractal pattern, generated from a binary, basic shape by three iterations. (**B**) Google Earth image of the settlement pattern in the Punjab region in 2018 at three levels of zooming-in (accessed from Google Earth Pro 7.1 on 21.09.2020).

While Euclidean geometries (such as triangles, squares or circles) are often used in architecture and planning to produce regular patterns, fractal geometry better describes many natural shapes with their irregularities and fragmentation^7^. Hence, fractal geometry, which implies heterogeneity, self-similarity, and hierarchical features, might be an underlying principle, or a general outcome of self-organisation, not only in nature but also in the spatial settlement forms created by societies^8,9^. Many built-up areas structurally follow a centrality principle with several hierarchical layers: a primary central place is surrounded by secondary centers, which are further surrounded by tertiary centers, and again by smaller settlements^10,11^. This principle results in a distribution in space that is self-similar through scales, as described by fractal patterns, such as the “Sierpinski Carpet”^12^ (Fig. 2).

In theory, fractal forms are built from the iteration of an initial point, curve, or surface by multiplying or dividing their outline or mass by a fixed quantity at each iteration step, and thus, look similar at different scales of magnification and/or resolution^7^. Fractals defined by iterated function systems display exact self-similarity. If fractal patterns appear approximately (but not exactly) identical at different scales, they are quasi-self-similar, contain small copies of the entire fractal in distorted and degenerate forms, and are mathematically described by recurrence relations. The weakest type of self-similarity is statistical self-similarity, as captured by the fractal dimension, a numerical measure which is preserved across scales^13^. The Sierpinski Carpet shown in Fig. 2A exemplifies an ideal, exactly self-similar pattern and has a fractal dimension of 1.89 (Hausdorff dimension^14^). The distribution of built-up area within a landscape (Fig. 2B), however, is subject to numerous factors, such as topography, population dynamics, land use, land prices, accessibility and the socio-political framework, which all potentially modify a theoretical self-organising principle, leading to distorted elements that may nevertheless appear similar (quasi self-similar) throughout the scales.

According to Garmestani et al.^15^, a pattern reflects “a function of processes in a complex system”. These authors explain the spatial distribution of cities by a slow dynamic process driven by the population and its survival needs. The landscape provides locations and critical resources (such as water for transportation, consumption, and irrigation) around which the human-ecological (urban) system self-organises. Settlement patterns are thus manifestations of the interplay between given biophysical features and dynamic processes (human activities) within the landscape. Therefore, the more homogeneous the conditions for self-organisation are, the closer the settlement pattern should be to exact fractal geometry.

In view of the above we therefore used a fractal theory framework to analyse the settlement pattern in a real-world landscape, the North Indian Punjab. More specifically, we ask how well the real-world settlement pattern represents the properties of the theoretical fractal structure of a Sierpinski Carpet. Such an analysis has to take account of an extended, continuous area (covering metropolitan to rural regions) and of the full range of settlement scales (small villages to million cities).

Our research approach deviates from conventional designs as it alternately applies inductive and deductive elements. To combine the *theory of fractal geometry* with *empirical data of the landscape*, we followed a stepwise approach, whereby the results of one step often become parts of the methodology in the next step. Briefly, our empirical data were *binary maps* of the study region, suitable for GIS-based geo-statistical evaluation. Using these maps, we developed a strategy to derive *landscape samples* that are representative across space and at multiple scales. Within these samples, we took GIS-based measurements of *spatial parameters*, such as settlement patch sizes, distances, and angles. We then analysed these parameters to characterize *pattern properties*, such as settlement size distribution, size classes and scales (by discontinuity analysis), spatial configuration, and self-similarity across space and scales. Finally, we applied observed spatial parameters to construct a *fractal model* and assess its fit to the real-world pattern.

Our approach is thus theory-driven (rather than data driven), and differs from both, existing urban studies that analyse urban form and function of individual cities, as well as studies of landscape ecology that address spatial relations of land cover patches with different biotic or abiotic properties. Understanding a large-scale landscape context as emerging from self-organizing principles may shed new light on development and transformation processes in general, and thus also reveal new approaches to govern these processes.

## Results

### Settlements show the same size distribution for different sampling methods and at different scales

**Figure 3:**
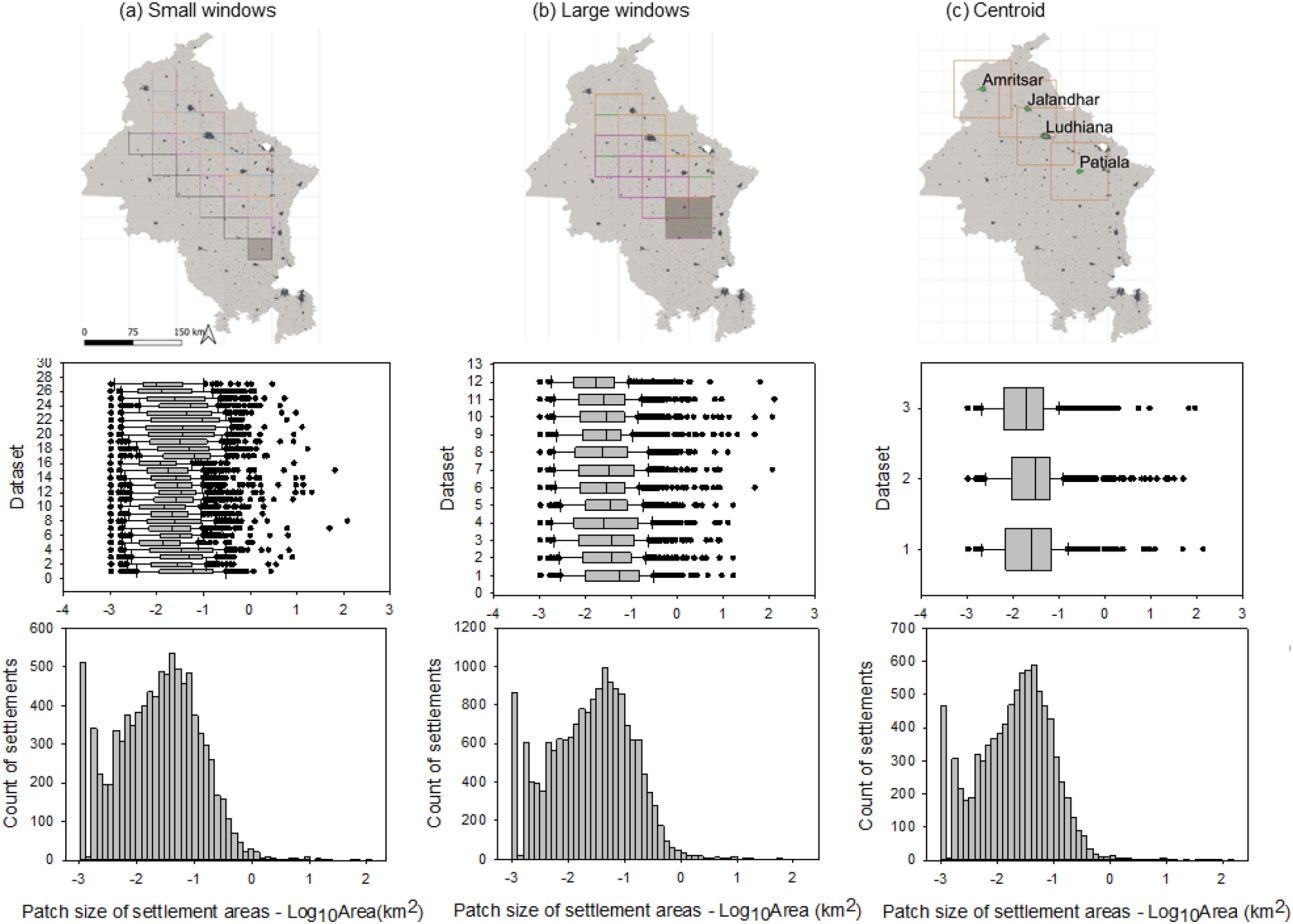
Settlement size distribution in the N-Indian Punjab. Maps, box plots and histograms of sampling used the moving window approach with (**a**) small and (**b**) large windows, and (**c**) the centroid approach. The small window generated 27 datasets, the large window 12 datasets, and the centroid sampling generated three datasets surrounding the mega settlements of Ludhiana, Patiala, and Jalandhar (Settlement binary map of 2016). The histograms aggregate the data shown in the box plots for each sampling procedure.

The elements of a highly regular pattern show similar size distributions irrespective of the sampling space and scale. This motivated the landscape sampling strategies based on square windows of different sizes (for details see methodology). The *semi-random moving window approach* started from a random point and captured similarities across the landscape by virtual flights, while the *centroid approach* took three out of four major cities as starting points and captured similarities across scales in buffer squares around them and their subordinate centers. The moving window approach covered a total of 9359 settlements (Fig. 3a,b), and the centroid approach (Fig. 3c) included 8760 settlements (2968 around Ludhiana, 2928 around Patiala, and 2864 around Jalandhar). Settlement sizes ranged from ca. 1000 m^2^ to 135 km^2^. Small settlements constituted the vast majority in the landscape, as evident from the low average size of 0.12 km^2^. Overall, 90% and 84% of the settlements were smaller than the average value in the centroid and moving window approach, respectively. The median value of 0.02 km^2^ was six times lower than the average value.

The distribution of settlement sizes within our sampling windows was consistent, irrespective of the sampling method and the scale of analysis/size of the windows (Fig. 3). The pairwise comparison of large moving windows and centroid approaches showed that only 7 out of 36 pairs were significantly different (*p* < 0.05; Table S1). That indicated a high degree of similarity in size distribution across scales, and a very homogeneous pattern over the entire region of analysis.

### Discontinuities in the size distribution of settlements reveal four size classes

**Figure 4:**
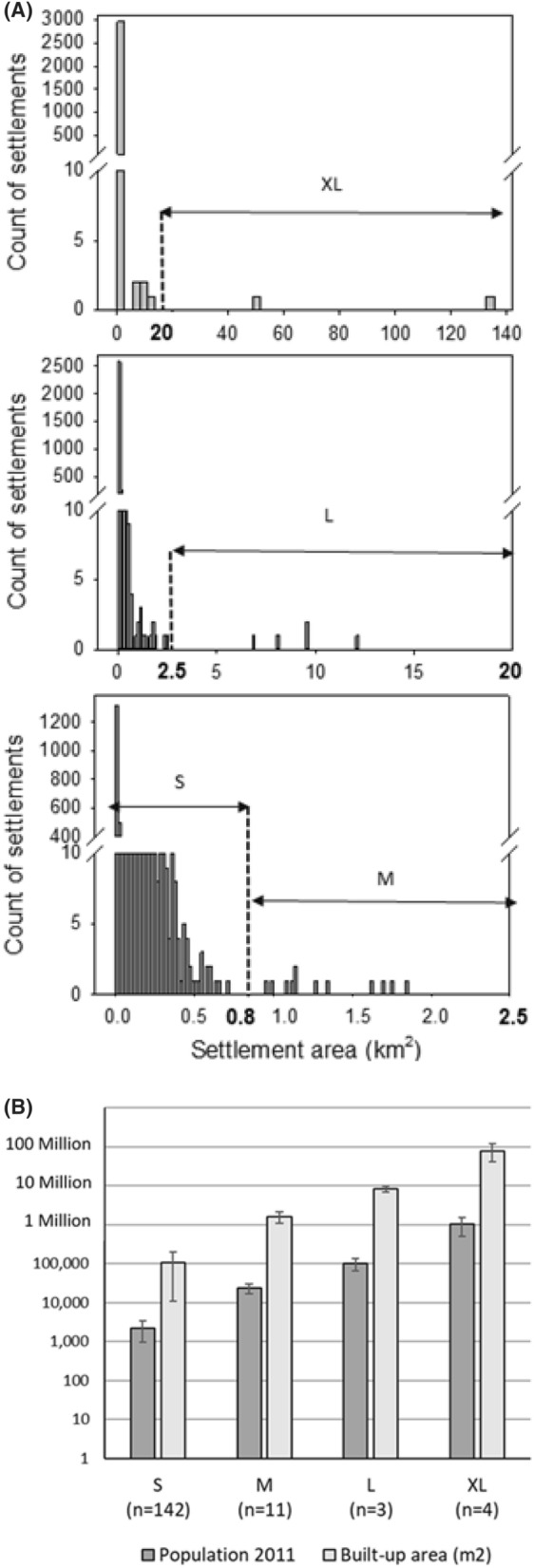
**(A)** Settlement size classification. Exemplary data from the centroid approach, Ludhiana sample (2968 settlements), N-India. Entire size distribution, showing the gap around 20 (km^2^), separating the XL settlements from the rest of the population. Size distribution truncated at 20 km^2^ showed the gaps around 2.5 km^2^ that delineates the L settlement from the lower size classes. Truncation at 2.5 km^2^ allows to distinguish medium and small settlements. **(B)** Siz e classification of settlements in the N-Indian Punjab by population data and by measured built-up area based on discontinuities in the distribution identified the same four size classes.

Discontinuities in size distributions indicate that the elements fall into different size classes, which correspond to scaling levels in a complex pattern. Based on the settlement size distribution, we detected four classes separated by gaps (Fig. 4A) around 20, 2.5 and 0.8 km^2^, where settlement patches were lacking. The resulting four size classes were defined as small settlements (S, < 0.8 km^2^), medium (M, 0.8–2.5 km^2^), large (L, 2.5–20 km^2^), and very large settlements (XL, > 20 km^2^). The XL class covered a wide range (from 20 to 135 km^2^) as it included the centroid centers Ludhiana, Jalandhar, and Patiala. Further sub-divisions, however, were not made due to the low number of settlements in this size class. The L class was clearly distinguished from the XL and M settlements, as well as the M class from the S cluster. The size classes were discretely distributed with wide gaps in Ludhiana and Jalandhar, while in the case of Patiala, many XL settlements of lower size range were found near the border to the Himalaya foothills and near water resources. M and S settlements were distinguished by smaller gaps but higher density (counts) of settlements. Especially the S class accounted for 80% of all settlements in the sample.

We used the database ‘citypopulation’ to compare this classification to the distribution of population data in the Ludhiana sample. The 160 sampled settlements also fell into four size clusters, whereby villages with less than 10,000 inhabitants constituted 89% of the sample (Fig. 4B).

In terms of Indian administrative units, Municipal councils and Nagar panchayats formed the medium-sized cluster with 10,000–50,000 inhabitants, as well as the large cities with populations of 50,000 to 250,000. Only the four centroid cities, all municipal corporations, had larger populations than that ranging widely from 300,000 (Patiala) to 1.6 million (Ludhiana). Population data correlated well (r = 0.9591) with the measured built-up area of the settlements. All settlements were assigned to the same class by both parameters. These findings validate our classification by measured built-up area.

The four spatial size classes were treated as scaling levels in subsequent analytical steps.

### Distance relations and angles vary at different scales but show high statistical similarity

**Figure 5:**
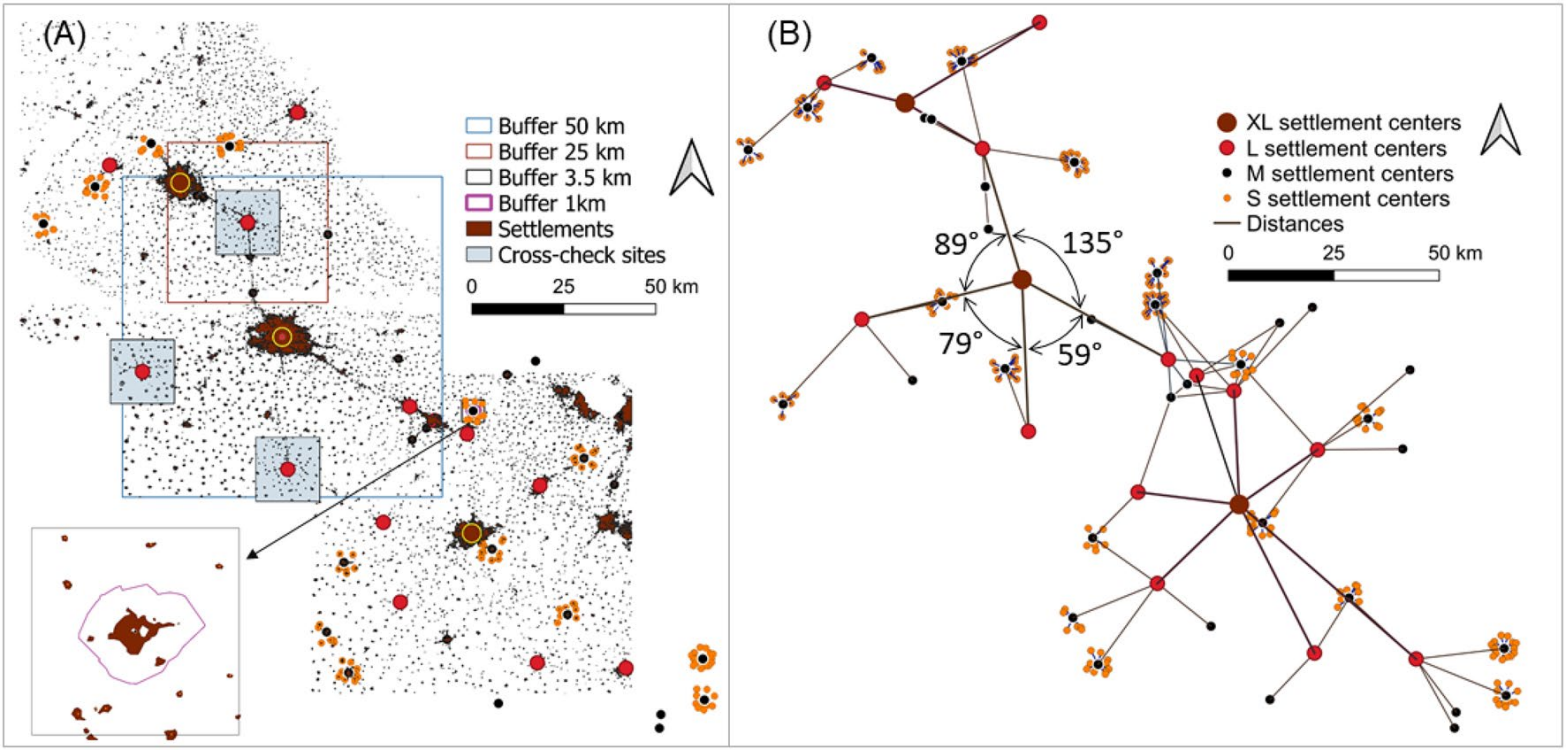
Three levels distance and angle assessments in the N-Indian Punjab. (**A**) Map with buffer sizes: 50 km, 25 km, and 3.5 km around XL, L, and M settlements; insert: buffer of 3.5 km with 1 km inner threshold around M settlements. (**B**) Hierarchy network: level 1 measurements from XL to L settlements, level 2 measurements from L to M settlements, and level 3 measurements from M to S settlements. Exemplary angle measurements are shown for one XL settlement (Settlement binary map of 2016).

Distances and angles between the elements of a pattern determine their spatial configuration. These parameters were assessed in representative landscape samples and evaluated statistically. Based on the class assignment we constructed a hierarchy model of the settlement network around the centroids (Fig. 5). The distances between neighboring settlements of the same size class were used to define the buffer zones for the measurements of distances and angles to the next lower size class (for details see methodology), whereby the angles depend on, or indicate the number of neighbours.

The four size classes allowed for three levels of analysis (Fig. 5). Level 1 referred to measurement of distances between XL settlements (Ludhiana, Patiala, and Jalandhar) and neighbouring L settlements in a 50 km buffer with n = 14 measurements. Level 2 referred to the measurements between L and M settlements in a 25 km buffer around 14 L settlements (such as Malkerkolta, Phagwara, Jagraon; total n = 52). Level 3 referred to the measurement of distances between M and S settlements in a 3.5 km buffer with an inner threshold of 1.6 km distance around the M center (n = 197 in 52 subsamples). Altogether, the geostatistical analysis was thus based on the measurement of 262 distances and 260 angles.

On average, distances were 33.8 km from XL to L, 18.5 km from L to M settlements, and 2.9 km from M to S settlements (Table 1). Average angles were 87.1 and 79.2 at level 1 and 2 (corresponding to 4 to 4.5 neighbours), and 34.6 at level 3 (10 to 12 neighbours). Median distance values were similar to the average distances at level 2 and 3, but differed at level 1. Average and median of angle measurements differed at all three levels.

**Table 1:** Descriptive statistics of distances (km) and angles (degrees) between settlements of different size classes in the Punjab of N-India.

	Distances (km)		Angles (degrees)	
	Average	Median	Average	Median
Level 1 (XL–L)	33.78	30.89	87.13	92.02
Level 2 (L–M)	18.45	19.31	79.22	84.4
Level 3 (M–S)	2.87	2.87	34.65	28.26

In a pairwise comparison of the three centroid samples (Table 2), the distance relations showed a high degree of similarity at all three levels with *p* values of 0.34 (level 1), 0.64 (level 2), and 0.59 (level 3).

**Table 2:** Statistic of distances and angles from the larger to next smaller size class at three levels in the Punjab region of N-India.

	*P*	*P* < 0.05
**Distances**		
XL–L	0.34	No
L–M	0.64	No
M–S	0.59	No
**Angles**		
XL–L		
Jalandhar versus Patiala	0.04*	Yes
Ludhiana versus Patiala	0.17	No
Jalandhar versus Ludhiana	0.26	No
L–M	0.75	No
M–S	0.96	No

*Differ from overall frequency at *p* < 0.05.

Angles centered at the three XL settlements showed statistical differences in one out of three pairwise t-tests, while the two other pairs were similar. At level 2 and 3, angles at L settlement centres to M centers were similar (*p* = 0.75), and there was no significant difference between mean angles of M to S settlements, (*p* = 0.96). In summary, the geo-statistical evaluation confirmed a very regular spatial configuration throughout the Punjabi landscape.

### The settlement pattern has a high degree of complexity and shows fractal features of self-similarity close to Sierpinski Carpet rules

The size ratio for the fractal modeling was derived from the average area of the settlement patches of successive classes (Table 3).

**Table 3:** Patch size ratio between settlements of different size classes in the Punjab of N-India.

Class	Average area (km^2^)	Sqroot (km) (side length)	Size ratio
XL	77.5	8.8	
L	11.3	3.4	XL/L: 2.6
M	1.7	1.3	L/M: 2.6
S	0.4	0.6	M/S: 2.0

#### Fractal dimension

The overall complexity of a fractal pattern is described by the fractal dimension (D_B_). Similar to fern leaf or snowflake structures, small copies (at local/low scale) repeat the features of the entire structure (at landscape/higher scale). Based on the size ratios determined in the geo-spatial measurements (Table 3) we calculated D_B_ for the three centroid samples around Patiala, Ludhiana, and Jalandhar (Table 4)^14,16^. With values of 1.96, 1.95, and 1.95 they prove high complexity. According to the list of Hausdorff dimensions^17^, the structure of the settlement pattern in the Punjab ranged between Sierpinski Carpet (D_B_ = 1.893) and Mandelbrot set (D_B_ = 2). They are thus not strictly self-similar but represent quasi-self-similarity, which means the patterns at smaller scales are slightly modified versions of the pattern at larger scales.

**Table 4:** Fractal dimension (DB) using box counting method in the three large buffers around the XL settlements of Ludhiana, Jalandhar, and Patiala in the N-Indian Punjab.

Box size (number of pixel)					
Buffer center	C1360	C453	C151	C75	D_B_
Patiala	1	9	81	616	1.96
Ludhiana	1	9	80	595	1.95
Jalandhar	1	9	78	575	1.95

#### Fractal projections

**Figure 6:**
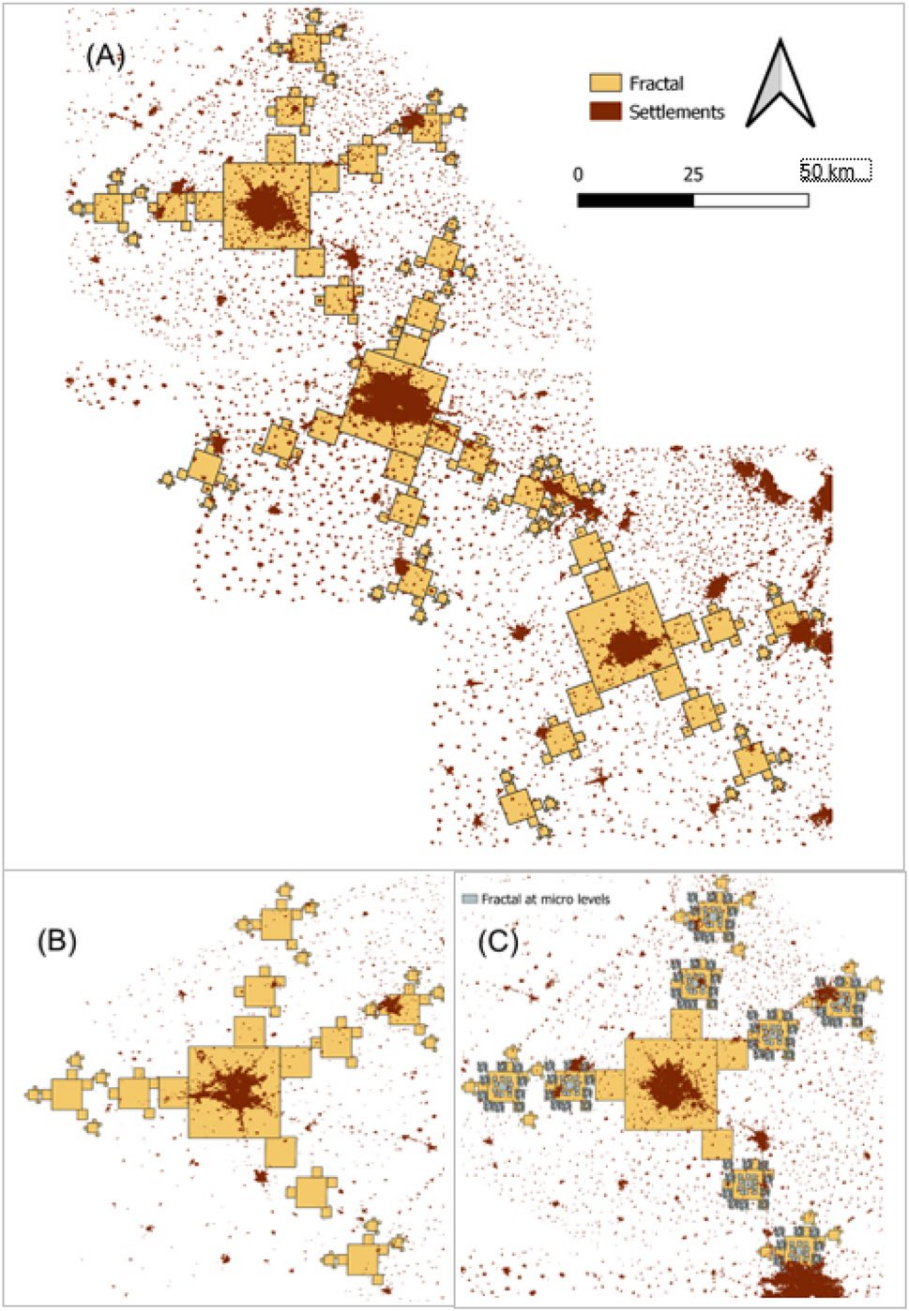
(**A**) Sierpinski Carpet fractals projected to the landscape, centered on the N-Indian mega settlements of Jalandhar, Ludhiana, and Patiala (Settlement binary map of 2016). The fractals were generated using Fractalopolis model at iteration 3 and (**B**) at Amritsar, (**C**) at micro level starting at iteration 2 in Jalandhar.

Finally, an attempt was made to derive a Sierpinski Carpet from the parameters characterized by the spatial configuration analysis above, and to apply the Fractalopolis model^18^ to assess its fit to the real-world settlement pattern. The Sierpinski Carpet shown in Fig. 2 is constructed on a square grid, that is a lattice with 90° angles and potentially eight nearest neighbors for a central patch. Other versions of the pattern can be built from triangular, penta- or hexagonal geometries. In our sample, the average number of subsets (that is, neighbouring settlements of the next lower size class) was 4 (range from 1 to 6) at level 1 and 2, and 10 (range from 6 to 16) at level 3. There was no statistic difference in number of subsets at levels 2 and 3 (*p* = 0.56 and *p* = 0.95, respectively). The size ratio between a center and its subsets was given by average size of the settlements in the respective class (Table 3). To generate our Fractalopolis model^18^, we selected the sample closest to the average values (Jalandhar with 4 subsets at level 1, and Phagwara with 4 subsets at level 2). Two three-step iterations were calculated for the four scales detected in our sample: Macro level represents the iterations from XL to L to M settlements, while micro level represents the iteration from L to M to S settlements. The structure established from the initial generator Jalandhar was finally shifted to the other locations, Ludhiana, Patiala, and Amritsar (Fig. 6).

At iteration 3 (macro level, Fig. 6A) 2/4 fractal squares matched roughly with the actual positions of large settlements around Jalandhar. Around Ludhiana and Patiala, 3/4 fractal squares matched after rotating the structure. However, only 1/4 of fractal squares matched the large settlement positions in case of Amritsar (Fig. 6B).

At micro level, the simulation of Sierpinski Carpet structure with 10 subsets did not show a match to the landscape pattern (Fig. 6C). Allover, the fractal structure failed to cover the evenly distributed and closely spaced small settlements.

## Discussion

### Hypothesis and approach

Our approach builds on the combination of three strands of research that are largely disconnected in the current urbanisation discourse. In urban geography and demographic statistics, studies on *systems of cities* are usually based on population figures and economic parameters^15,19^. They understand urbanisation as a process that concentrates people and economic opportunities in ever-growing cities. Earth observation and land use science apply remote sensing products to map *urban footprints* in terms of built-up land cover^5^, and show urbanisation as spatial sprawl of cities. Systematic cross-evaluations between those two fields, as well as analyses of *patterns at the landscape scale* that integrate rural surroundings, are scarce^20–22^. Even more disconnected is the research on pattern formation grounded in complexity science. *Fractal theory*, for example, has been applied to various examples of physical phenomena^23,24^ or biological morphogenesis^25,26^. It has also been applied as tool for spatial analysis of urban agglomerations since the 1990s^27,28^, but mainly focusing on individual large cities^29–31^. Later on, some authors adopted it as a potential tool for city planning^9,12,18^. However, it has not been applied to settlement systems in extended landscapes including small settlements, as attempted here. In more general terms, the analysis of complex systems has shown that self-similar fractal patterns can arise from self-organisation^26,32^. In this paper, we thus aimed at characterizing the spatial pattern of settlements in an extended landscape under a theoretical framework that may allow at least speculations towards the underlying organizing principles.

### Systems of cities, urban footprints and mixed approaches

The trivial terms city, town, and village typically imply a size classification of settlements, that has been formalized in many different national and international systems to support administrative hierarchies and development policies. The Census of India^1^, for example, defined six classes by population numbers, with (arbitrary) cut-offs at 5, 10, 20, 50, and 100 thousand inhabitants, and distinguished administrative designations such as Municipal Corporation, Municipal Council, Nagar Panchayat, Census Town and Village that reflect size and rank relations. Researchers developed theoretical underpinnings such as Central Place Theory^10^, or power laws of city size distribution (rank size rule, Zipf’s law^33^). Villages of less than 10.000 inhabitants were not included in those analyses. The definition of city size classes has a crucial impact on the description of systems of cities. In our study, we derived the thresholds for size classes and scaling levels from endogenous discontinuities in the distribution to avoid arbitrary cut-offs. The non-linearities, discontinuities, and ‘clumping’ frequently observed in urban (socio-economic and demographic) features gave rise to the idea that self-structuring processes, as described for ecosystems^34^, may also contribute to the development of city systems^8,15,33^. This thought is taken further in our study by linking the principle of self-organisation to the physical outcome of fractal structures.

The mapping of urban sprawl to document urbanisation became a growing field of research with the increasing availability of high-resolution satellite images. These studies addressed metropolitan regions^3,5^ as well as local or global scales^4^. They usually focus on quantifying the conversion of land use types, in particular from agricultural to built-up land, and limit themselves to the spatial analysis.

Demographic and spatial data were integrated, for example, in the development of the ‘IndiaCities’ database^20^ to overcome inconsistencies in the classification system used by the Indian government, which tends to overlook urbanisation in secondary and smaller towns^35^. Current initiatives to characterize urbanisation patterns in other parts of the world follow up on this approach (eGeopolis, Africapolis^36,37^). Spatial information in these initiatives also helps to fill gaps where statistical data are lacking or incomplete.

Most of the literature on city size distributions and its inherent discontinuities still relies on population data^15^. A critical comparison of the rank size distribution of cities according to population data with a ranking based on settlement sizes determined from remote sensing was presented by Bajracharya and Sultana^21^ for Bangladesh. They found significant deviations from Zipf’s law, and argue for a better integration of spatial data in urban development policies. We also crosschecked these parameters in our approach. When population data extracted from the database ‘citypopulation’ were analysed for discontinuities in the same way as the settlement patches, the resulting size classes were congruent. We considered this a validation of our classification approach.

### Patterns at the landscape level

Few studies have characterized settlement patterns at the landscape level, including settlements below the town or city threshold (Esch et al.^38^; Esch et al.^39^; Cantteneo et al.^22^; and Li et al.^40^). They are similar to our study in some aspects, but clearly different in others, and thus merit a more detailed comparative discussion.

Esch et al.^38^ pointed out that the dimension and structuring behind urbanisation processes comprises a spatial continuum stretching from megacities to sparsely inhabited, rural areas. Spatial metrics are an effective tool to describe the spatial structure and fragmentation of landscapes, but they have limitations when assessing the relevance and relations within a hierarchical system or network of objects or patches. Esch et al.^38^ developed a big data approach applying spatial network analysis to binary settlement maps at regional, national, and global spatial scales. In this methodological concept and technical procedure multiple geospatial metrics were complemented by a selection of various attributes, weights, and relevance indices of interest to capture the importance of a given node in the network. Centrality and hierarchies were derived empirically from these measures, whereas in our approach we defined them prior to the spatial measurements, by defining size classes and scaling levels based on theoretical fractal patterns. Our analysis builds on the binary maps developed by DLR^39^, and is inspired by the measures applied in Esch et al.^38^, such as distances between centroids, or number of connections within a defined search buffer (in our analysis captured by number of neighbors or angles). However, our metrics are calculated “manually” and thus we had to rely on smaller but, as our results show, still representative landscape samples and subsamples. Automated computing methods would allow increasing the *n* and thus improve the validation of our hypotheses. Nevertheless, the evidence for self-similarity in our results was strong.

Concepts of centrality and hierarchies in a rural–urban continuum were taken further in the recent work of Cattaneo et al.^22^. They endogenously identified functional areas around urban centers of reference, designated as “rural catchment areas”. Similar to our study, this method also avoids arbitrary classification thresholds and works over a wide range of scales.

**Figure 7:**
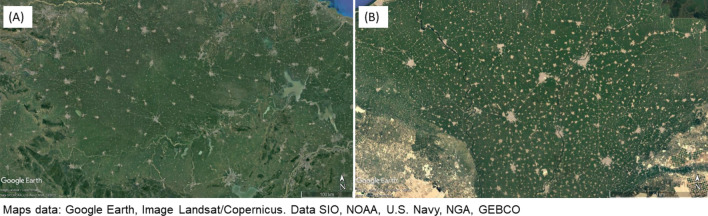
(**A**) Settlement pattern of the North China Plane, and (**B**): of the Nile delta in Egypt (Source: Google Earth Pro 7.1, accessed on 08.02.2021).

Li et al.^40^ characterized settlement structures in China from a land cover change perspective. Similar to our sampling method, they defined parameters that relate to the degree of clustering, using a window moving around a given central window. From spatial parameters within the windows they derived parameters for cluster density and cluster size, in addition to the share of built-up area within the sample, yet at a fairly low scale with window sizes of 2 km^2^, or 36 km^2^ for a cluster. They also emphasized the predominance of village landscapes as compared to urban settlement systems. Their results challenge the research focus on large cities, as small incremental growth in small settlements, which often remains unnoticed, often exceeded the rapid and profound transformations in urban agglomerations in terms of net area. They concluded that a more integrated landscape approach that includes rural regions is needed to fully understand dynamics of urbanisation. Their approach, however, though methodically similar to ours, is purely empirical and not grounded in theories such as hierarchical networks or fractal geometries. As argued in our study, these authors pointed out the limitations of studying only the conversion of non-built-up to built-up land, as well as describing systems of cities by only demographic statistics (population, administrative status, economy). Much in line with our approach, Li et al.^40^ used spatial pattern indices for the characterization of settlement systems that span the entire spectrum from rural to urban landscapes. In their system, the intensive agricultural area of the North China Plane was characterized as “sparse and dense village landscapes”, in which settlements are densely clustered, but the clusters themselves are smaller. They claim that this pattern emerged exactly because of the agricultural character of the region.—Actually, the settlement pattern of the North China Plane has striking similarity to the Punjab (Fig. 7A), and it would be interesting to also apply our approach to that region which has a similarly large alluvial area with intensive irrigation agriculture. Another similar settlement pattern is found in the Nile delta (Fig. 7B). Fractal structures confirmed in these locations would support the argument that this geometry results from self-organisation, and is the result of an evolution to maximize efficiency in agricultural production functions under certain boundary conditions, such as flat topology with fertile land, long history of small-scale agriculture, high population density, catering a nearby megacity. The impact of the different factors would warrant deeper investigation.

### Fractal theory and city planning

According to Mandelbrot^7^ “A fractal is a mathematical set or concrete object that is irregular or fragmented at all scales”. That certainly applies to the Punjab landscape analysed in this study where fractal features were confirmed by the fractal dimension (D_B_ = 1.95) and a high degree of statistical self-similarity in the settlement size distribution and landscape metrics. Similar results (D_B_ = 2) were reported, for example, for the distribution of galaxy clusters^24^. In our study, however, projections of the modeled Sierpinski Carpet on the Punjab landscape did not fully capture the settlement structure across all scales. While quasi-similarity was indicated at higher scales (in the hierarchy relations between XL, L, and M size cities), the number of subsets and the spatial distribution of small settlements at the lowest level deviated from the fractal geometry. Instead, the space in between the hierarchically organized larger settlements seemed to be filled with evenly scattered, small villages (Figs. 2, 5A, 6). This may represent a different organizing principle onto which the hierarchical network with its fractal features is superimposed. Phase transitions^41^ or regime shifts^42^ may be theoretical concepts to further analyse this phenomenon.

We adopted the fractal modelling from Yamu and Frankenhauser^11^ who developed a multiscale, multifractal setup to predict and manage urban sprawl. In their simulations, the authors also considered geographical and economic factors, such as monthly, weekly, and daily visited facilities and leisure amenities. That proved to affect cities sizes and manifest the discontinuous structure in city size distribution^35^. The study of Frankenhauser et al.^18^ aimed at optimizing urban form and function for sustainability by limiting negative effects of urban sprawl. As it originally targeted two medium-sized French metropolitan areas it addressed regional to neighbourhood scales in a Western context which is fundamentally different from our study region. The potential of multifractal modelling for improved city planning was substantiated in subsequent publications^18^, but so far it was never applied as an analytical tool, at larger scales, or in rural areas worldwide.

For the foreseeable future urbanisation will continue worldwide and will further expand into rural regions. Hence there is a need to better understand the urbanisation processes not only in urban, but also in rural and transitional regions, and develop strategies to make urban systems more sustainable at national to global scales^39,43,44^. Theory-based perspectives may enrich the discourse currently dominated by empirical and planning approaches.

## Conclusions

Starting from the striking settlement pattern in the Indian Punjab, we explored how well it complies with the fractal geometry of a Sierpinski Carpet, and could show substantial evidence for self-similar, fractal features in the real-world landscape. Since self-organizing systems often produce fractal patterns in nature, we conclude from our findings that also in the evolution of settlement structures self-organising principles act in parallel to human planning. Our study is certainly not sufficient to establish specific causalities or mechanisms, but similar fractal patterns evolved in other places of the world. Fractal forms may constitute a nature-based alternative to established planning paradigms, and a comparative analysis of different case studies may yield further insights to the interplay of the governing factors.

We combined elements of inductive and deductive research approaches, aiming to gain basic knowledge, rather than provide application oriented problem solutions. This may nevertheless raise the awareness of planners and policy makers for inherent system properties and organizing principles. Employing such principles in urban design and regional development rather than overriding them may result in more sustainable rural–urban transformations.

## Materials and methods

### Study region

Located in a vast fertile plane, the Punjab region stretches across North Pakistan and North India. On the Indian side sits the bread basket of India’s National Capital Region around Delhi, as well as one of the country’s most urbanised and developed regions besides its metropolitan areas^6^. Irrigated agricultural fields and settlements of different size dominate the landscape, while other land uses are negligible (Fig. 1). The Punjab is thus readily represented by *binary data*, with only built-up and non-built-up land cover (Figs. 1, 2). Settlements in the Punjab range from cities densely populated in the millions to villages of barely 1000 inhabitants, with apparently regular, hierarchical spatial distribution and apparent self-similarity across scales (Fig. 2B). In the this study we refer to settlements as an overarching term for villages, towns, and cities as patches of built-up area used for housing and business activities, irrespective of their size, population, or administrative status.

### Settlement binary maps

We collected settlement binary maps of 2015 at 30 m resolution from Liu et al.^43^ and of 2016 at 10 m resolution from the DLR (Deutsches Zentrum für Luft- und Raumfahrt—German Aerospace Center). We used the settlement binary map of 2015 at 30 m resolution for analysing the mathematical parameters of settlement pattern, and the higher resolution of the 10 m settlement map for validating the spatial fractal pattern.

The binary raster maps consisted of built-up (code = 1, black) and non-built-up (code = 0, white) classes. The raster files were extracted using the boundary polygon of Punjab and Hayriana states. Only built-up areas of at least 1000 m^2^ were considered as settlements whereby size was estimated using Google Earth images for areas of more than three houses, which formed a village or a hamlet. “Lonely” pixels in settlement binary map in 2015 were reduced by assigning them to the value that more than half of their eight neighbouring pixels showed (half dominance majority method^45^).

### Landscape sampling

Sampling was necessary to reduce the overall amount of data to a range that could be processed with available computer (PC) capacity. We defined square windows as landscape samples, and applied two sampling methods: the “semi-random moving window” and the “centroid window” approach with multiscale sampling.

#### Semi-random moving window

A square grid was adjusted such that a grid cell covered at least one large settlement and at least three surrounding smaller settlements. A grid size of 1312 km^2^ (i.e. a square of 36.2 km side length, defined as small window) fulfilled this condition. This net covered the entire study region by 15 rows and 10 columns. Four small windows were aggregated to a large window (5248 km^2^, 72.4 km side length; Fig. 3). We selected a random point to begin a ‘virtual flight’ of the small moving window in the direction of the longest straight path in northwestern direction. From there, paths were extended to the north and south until in total, we examined 27 small windows (five virtual flights, consisting of five to six windows each). Following the same flight pattern, the large windows had an overlap of 25%, and three flights, each consisting of four windows were examined leading to 12 large windows in total.

#### Centroid window

We selected the four largest settlement polygons in the Punjab region as centroids, i.e. the cities of Ludhiana, Patiala, Jalandhar, and Amritsar, which in 2011 had populations > 1 million^1^. Based on the smallest distance between these settlements, four buffer squares of 50 km side length were generated from the center point of each settlement, representing the highest spatial scale level. Three of those squares were used to measure parameters of the settlement patterns, while one (Amritsar) was reserved for subsequent validation. Within the 50 km buffer square, buffers of 10 km and 2 km side length were generated around large and medium-sized settlements to represent lower spatial scale levels (Fig. 3).

### Size distributions and discontinuity analysis

The different sampling sets (i.e. 27 small windows, 12 large windows, and 3 centroid windows) provided the database for visualizing the size distribution by box plots and histograms (Fig. 3). This measure was used to validate if our sampling was representative of the entire landscape, to assess the regularity/self-similarity of the pattern, and it provided the basis for size classification by discontinuity analysis^33^, where different classes are distinguished by gaps in the distribution (S, M, L, and XL settlements separated by gaps at 0.8, 2.5, and 20 km^2^, respectively).

**Figure 8:**
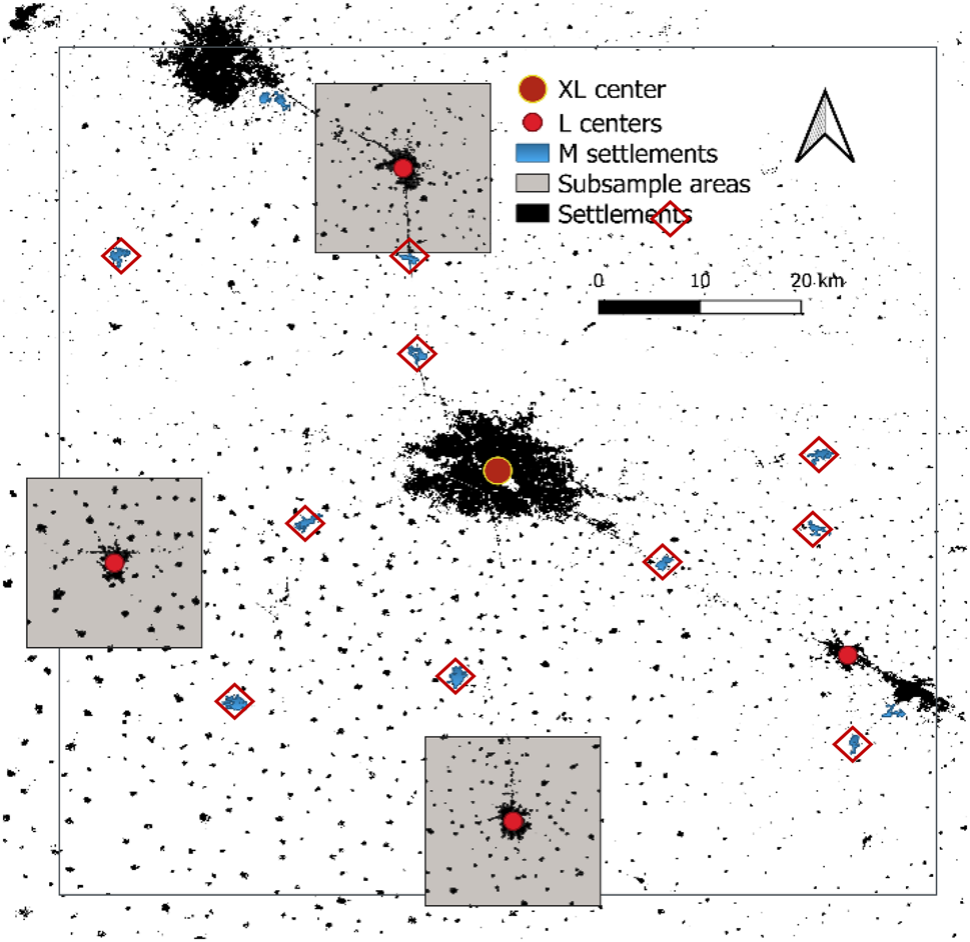
Subsample for comparison of mapped settlement sizes with data extracted from CityPopulation database for the Punjab in N-India. M settlements included in the analysis are marked by red diamonds (Settlement binary map of 2016).

To compare this classification to population data, we extracted from the publicly available database ‘citypopulation’ the population count in 2011 for all XL, L, and M-settlements within the centroid sample around Ludhiana, and for the S-settlements in three subsamples around Malkerkolta, Phagwara, Jagraon (Fig. 8). Altogether 160 settlements were included.

### Geo-statistical evaluation of distances and angles.

To obtain geo-spatial metrics we used QGIS 3.12. ArcGIS 10.3 was employed to measure distances, and (after conversion of the maps to .png format) ImageJ^16^ to determine angles. The metrics were recorded between settlements of a class and its nearest neighbours of the next lower class within a defined square buffer, and this computation was repeated at all scale levels. The buffers were derived from the distances within the same size class (Fig. 5A). The minimum distance between the four XL centroid centers Ludhiana, Jalandhar, Patiala, and Amritsar was 55.2 km. Hence, the buffer zone level 1 was rounded to 50 km side length. Likewise, the rounded minimum distance between L settlements of 25.3 km defined the buffer zone level 2 at 25 km around the L centers. However, due to high density and scatter of medium and small settlements, this strategy could not be applied at the lower levels. Small settlement patches close to the medium centers were often sprawl areas rather than individual villages, as evident from the crosscheck with secondary data (‘citypopulation’). Therefore, the buffer radius for level 3 measurements was finally defined by an inner threshold of 1.6 km and an outer limit at 3.5 km (Fig. 5A).

Representative samples were selected around M centers which were not located close to L or XL centers, not in a line of road, and including more than one S settlement.

### Statistics

We employed t-tests and pairwise t-tests in SigmaPlot to assess the similarity of size distribution, distances, and angles in the different samples at *p* < 0.05. Thereby in-significant differences between the moving windows and the centroid windows would indicate a regular settlement pattern throughout the region, while the similarity at different scaling levels (small and large windows) would indicate a high degree of fractal self-similarity in center-periphery relations.

### Fractal dimension

Among the mathematical descriptions of fractals, the fractal dimension (D_F_) is a common measure for the degree of self-similarity and complexity of a pattern. As a frequently cited example, the Sierpinski Carpet is initiated by a square, which is subdivided in the first iteration step by a 3 × 3 grid to generate 8 surrounding subsquares, each with 1/3 side length of the initial square and repeating the original pattern. At the next iterations, the same rule is applied to each subsquare, generating the pattern shown in Fig. 2A. After k iteration steps, the side length is 1/3 k. Hausdorff (14) defined this fractal dimension (D_F_) as:

$${\text{D}}_{{\text{F}}} = {\text{Log N}}/{\text{Log }}\upvarepsilon$$

where N denotes the new parts created each iteration and ε refers to the size ratio. For the example of the square Sierpinski Carpet above (Fig. 2B), this results in:

$${\text{D}}_{{\text{F}}} = {\text{ Log8}}/{\text{log3 }} \approx { 1}.{89}.$$

According to Hausdroff^14^, D_F_ ranges from 0 to 3, where a value of 0 represents points, 1 describes lines, 2 denotes surfaces, and 3 refers to volumes. Beyond the common understanding of one- two and three-dimensional objects, however, the fractal dimension can assume non-integer values. The range that D_F_ can theoretically assume in our two-dimensional settlement patterns is between 1 and 2. However, in real-world patterns it is not always possible to define the number of new parts for each scale, or the number of iteration steps, and thus the k value to determine D_F_. Therefore, DF is often indirectly estimated as the equivalent D_B_—a box counting dimension^13^. In this method, the fractal dimension is calculated by counting the number of boxes covered by the fractal pattern within a superimposed grid. This counting is repeated for different grid sizes to derive the fractal dimension.

To estimate the degree of self-similarity in the Punjab settlement pattern, we determined D_B_ for the three landscape samples around Ludhiana, Patiala, and Jalandhar that were generated in the centroid window approach (Fig. 3B). We exported the settlement maps within the square buffers as black and white image files (png format) and measured the fractal dimension (D_B_) using the box counting tool in ImageJ software^16^. To avoid a miscalculation of box counting dimensions caused by data scatter^14^, we applied the first grid box size covering the entire image. The other grid box sizes and the amount of size sets were defined according to the size ratio between the iteration steps. Fractal dimension D_B_ was measured by the equation:

$${\text{D}}_{{\text{B}}} = {\text{ lim}}\upvarepsilon _{{1}} \to 0\left[ {{\text{log N}}{\upvarepsilon /}{\text{log}}\upvarepsilon _{{1}} } \right]$$

where N: the number of boxes containing settlement, ε1: box size or scale.

If the values determined for the three samples are similar, the patterns have a similar level of complexity.

### Fractal projections

Finally, we crosschecked how well the actual settlement pattern at the landscape level matches a theoretical Sierpinski Carpet. To this end we applied the Fractalopolis model^11^ to generate fractals using the Sierpinski Carpet rule with three iteration steps. The sizes of initiator and sub-centre were estimated from the ratio between settlement size classes, median angles, and average number of sub-centres, as determined in the geo-statisical analysis.

The size of the fractal under iteration by equation:

$${\text{Ln}} = {\text{Ln}} - {1}\left( {{1}/{\text{Rn}} - {1}} \right)$$

where L is size of fractal at n iteration, R is size ratio.

Those fractals were overlaid to the real-world settlement patterns. We used the Jalandhar sample to generate the fractal structure, and then slided it across the study region (until the center was positioned at the XL centroids) and projected it to lower scales, aligning the axes to the L settlements (Fig. 6). This qualitative assessment was supported by geo-statistic outputs, thus roughly evaluating the match of the theoretical versus the real settlement pattern.

## Supplementary Information

Supplementary Information.
