# Supplementary material for: Spatial patterns of urbanising landscapes in the North Indian Punjab show features predicted by fractal theory
Source: Sci Rep. 2022 Feb 2;12:1819. doi: 10.1038/s41598-022-05906-4 (PMC8810886; doi:10.1038/s41598-022-05906-4)
Supplement: Supplementary file 1 — Supplementary Information. [file 41598_2022_5906_MOESM1_ESM.pdf]

Supplementary information

Spatial patterns of urbanising landscapes in the North Indian Punjab show features predicted by fractal theory

Thanh Thi Nguyen [a*], Ellen Hoffmann [a,], Andreas Buerkert [a]

[a] Organic Plant Production and Agroecosystems Research in the Tropics and Subtropics (OPATS), University of Kassel, Steinstr. 19, 37123 Witzenhausen, Germany

[*] Corresponding author, e-mail: thanh.nguyen@uni-kassel.de

Supplementary Table

Table S1. Comparison of moving window and centroid sampling approaches, p-value in t-test the count in in three flights (each four samples) at large window level and three centroids Ludhiana, Patiala, and Jalandhar in Punjab regions.

Moving window (Large windows)		Centroid		
		Ludhiana	Patiala	Jalandhar
Flight 1	Sample 1	0.016*	0.016*	0.017*
	Sample 2	0.032*	0.032*	0.033*
	Sample 3	0.069	0.069	0.073
	Sample 4	0.087	0.087	0.092
Flight 2	Sample 1	0.057	0.057	0.041*
	Sample 2	0.057	0.057	0.061
	Sample 3	0.051	0.051	0.055
	Sample 4	0.065	0.065	0.069
Flight 3	Sample 1	0.168	0.168	0.179
	Sample 2	0.173	0.157	0.168
	Sample 3	0.133	0.122	0.131
	Sample 4	0.066	0.064	0.069

*Differ from overall frequency at $p<0.05$
